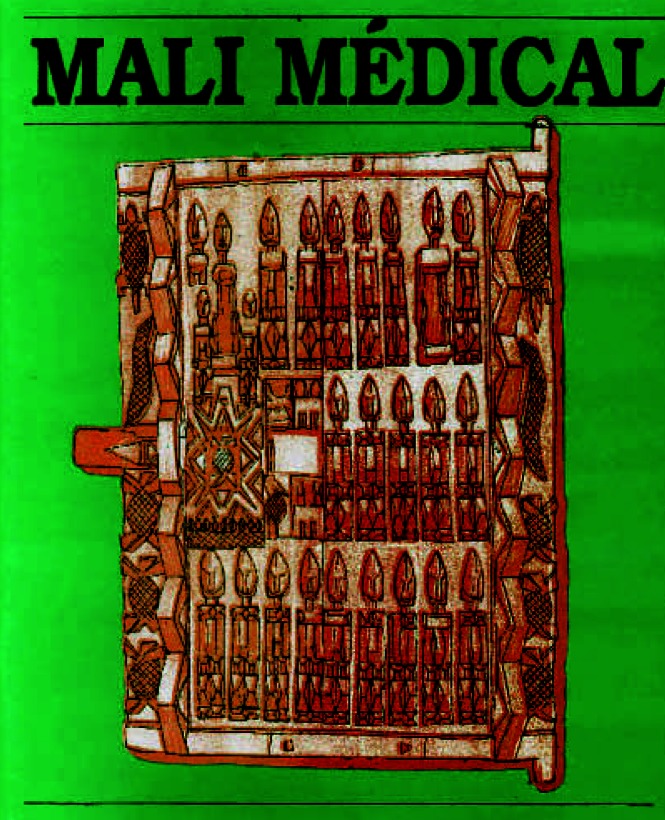# *Mali Médical* Goes Global

**Published:** 2007-05

**Authors:** Tanya Tillett

Since 2005, *EHP* and other members of the African Medical Journal Editors Partnership Program (AMJEPP) have worked with counterpart African journals to increase the latter journals’ capacity and reach. Now *Mali Médical*, the African partner journal for *EHP* and the *American Journal of Public Health*, has taken a big step forward in getting its published research out to a worldwide audience by becoming the second of the African AMJEPP journals to be accepted for indexing in MEDLINE, the essential database for far-reaching distribution of biomedical information. *African Health Sciences*, the Ugandan partner journal for *BMJ*, was indexed prior to the formation of the AMJEPP.

Due to the sustainability and capacity challenges typically faced by developing nations, the African journal partners have been hampered in their efforts to disseminate essential research information internally as well as internationally. The AMJEPP, currently funded by the National Library of Medicine and the NIH Fogarty International Center, pairs these journals with established journals in the United States and the United Kingdom that can offer guidance, training, and expertise (see “Global Collaboration Gives Greater Voice to African Journals,” *EHP* 113:A452–A454 [2005]).

Thomas J. Goehl, *EHP*’s former editor-in-chief and one of the architects of the AMJEPP, sees MEDLINE acceptance as a highly promising opportunity for *Mali Médical* to move beyond the foundation it has established in local African countries and increase its presence internationally in biomedical research. “The editors and editorial board of *Mali Médical* have worked very diligently in developing the journal into one that has become a focus for the medical community in many francophone countries in Africa,” Goehl says. “With the inclusion of the journal in MEDLINE, the rest of the world will now have much easier access to the first-rate articles being published by *Mali Médical*.”

Hui Hu, *EHP*’s international editor, agrees that making *Mali Médical* searchable on MEDLINE will allow it to be much more visible to the international audience. “This is a milestone step to the international community for *Mali Médical*,” she says.

Adding to this increased visibility is *Mali Médical*’s recent inclusion in the International Standard Serial Number (ISSN) Register, the world’s most comprehensive and authoritative registration source for the identification of serial publications. This will allow the journal to be cited, abstracted, and indexed more accurately.

Keeping in step with *EHP*’s ongoing mission to make environmental health science literature available to the largest audience possible, especially to those in developing countries, the journal joined Online Access to Research in the Environment (OARE) in January 2007. An international public–private consortium sponsored by the UN Environment Programme and Yale University, OARE provides web access to more than 1,000 scientific journals to developing, low-income countries for free or minimal cost. For more information on OARE, visit http://www.oaresciences.org/.

## Figures and Tables

**Figure:**